# The Impact of Face Masks on the Emotional Reading Abilities of Children—A Lesson From a Joint School–University Project

**DOI:** 10.1177/20416695211038265

**Published:** 2021-08-19

**Authors:** Claus-Christian Carbon, Martin Serrano

**Affiliations:** Department of General Psychology and Methodology, University of Bamberg, Bamberg, Bavaria, Germany; Taylor Ranch Elementary School, Venice, Florida, United States of America

**Keywords:** face masks, children, COVID-19, emotional states, STEM, intersectional school–university projects

## Abstract

Wearing face masks has become a usual practice in acute infection events inducing the problem of misinterpreting the emotions of others. Empirical evidence about face masks mainly relies on adult data, neglecting, for example, school kids who firmly are dependent on effective nonverbal communication. Here we offer insights from a joint school–university project. Data indicate that emotional reading of 9 to 10 years old pupils (*N* = 57) was similarly impaired as adults on an overall performance level, but that their selective performance on specific emotions was quite different. Kids showed extreme problems in reading the emotion disgust, strong effects on fear and sadness, and only mild effects on happiness, but also even better performances for emotional states anger and neutral when faces were masked. This project did gain not only relevant data about children’s perception but also made clear how fruitful seriously conducted school projects can be to encourage the interest and commitment for Science, Technology, Engineering, and Mathematics (STEM)-relevant topics.

In situations of acute infection events, wearing face masks is an effective measure to reduce the risk of being infected when combined with other hygienic measures such as social distancing and handwashing (Chu et al., 2020; Esposito et al., 2020; Verma et al., 2020). In extreme cases such as epidemics, wearing masks is a daily and ongoing practice and affects most places where people from different households gather together for an extended period. This leads to a long list of needed transpositions and adjustments, starting from well-thought hygienic and replacement concepts of face masks to the correct handling, dressing, and undressing of masks. Due to the mere physical occlusion of prominent and quite informative parts of the face (about 60% of the facial area, see Carbon, 2020), converging study results report problems on several dimensions: Identifying persons (e.g., Carragher & Hancock, 2020; Freud et al., 2020), understanding acoustic messages (e.g., Porschmann et al., 2020), and forming impressions about further person-related variables such as attributing trust (Marini et al., 2021). Emotional reading is also hardened by wearing face masks, at least as long as nontransparent, standard masks are used (Marini et al., 2021). Carbon (2020) was the first to substantiate the everyday problems in recognizing facial emotions people reported during the first wave of the COVID-19 Pandemic, back in May 2020: Not only the recognition performance of emotional reading decreased, but the participants also confused several emotions, especially disgust (confused with anger), happiness (with a neutral emotional state), and anger (with disgust, neutral, and sadness). The participants also reported lower degrees of confidence in their emotional reading capabilities (Carbon, 2020). Altogether, this indicates a clear handicap for nonverbal communication when face masks are used. These results are mirrored by subsequent research and analyses (e.g., Mheidly et al., 2020) and are supported by practice reports from areas where emotional reading is pivotal, for instance in psychiatric and psycho-therapeutical practice. Masks may particularly diminishing the perception of positive emotions (Nestor et al., 2020), especially expressed by happy faces which are mainly indicated by a toothy grin. The impact of masks on negative emotions is less clear, but it seems that masks do not specifically increase the feeling that the mask wearer shows negative emotions (Carbon, 2020; Marini et al., 2021).

The scientific database of the impact of wearing masks on children’s face processing performance is meanwhile thin although there are some first examples available (e.g., about face recognition performance, see Stajduhar et al., 2021). Regarding research on the impact of masks on the ability to read emotions from masked faces is mostly lacking. The main reason for this overall low number of studies might be that during several lockdowns during the COVID-19 Pandemic, empirical studies with school kids facing heavy hygienic requirements are technically hardly feasible. A rare empirical study of this kind was provided by Ruba and Pollak (2020) who employed 7 to 13 years old school kids using the Japanese and Caucasian Facial Expressions of Emotion (JACFEE) face database (for reliability data, see Biehl et al., 1997; Matsumoto & Ekman, 1988). The authors used frontal depictions of stereotypical facial configurations showing three different negative emotions: sadness, anger, and fear. They employed pictures of two persons showing each emotion without a mask (original depictions from JACFEE) plus two other persons showing each emotion with graphically added masks—additionally, they employed a condition where sunglasses had been graphically added. Children were more accurate in inferring others’ emotion when faces were unmasked—this showed up with a large effect size, Cohen’s *d* = 0.73 (see Cohen, 1988). Despite this clear effect, the authors summarized their findings that children “may not be dramatically impaired by mask wearing during the COVID-19 pandemic” (Ruba & Pollak, 2020, p. 9), by focusing on the above-chance level performances and due to the fact that the participants were not less handicapped in reading emotions when face masks covered others’ faces than when covered by sunglasses. In the case of face masks, they concluded that the eye region is sufficient in dissolving the targeted emotions, which mirrors recent results from presenting eye regions only (Schmidtmann et al., 2020). Nevertheless, the study by Ruba and Pollak (2020) is rather limited for making solid inferences on the recognition ability of emotions in masked faces per se because only three emotions were tested, and all of these emotions were negative emotions (sadness, anger, and fear). Furthermore, the authors explicitly claimed that these emotions were selected because “adults tend to fixate predominantly on the eyes for these facial configurations, rather than other parts of the face” (Ruba & Pollak, 2020, p. 3) which reduces the relevance of these stimuli for studies on the impact of face masks as they exactly do *not* cover the eyes region. Interpreting the data of the Ruba and Pollak study is further aggravated as diverse persons showed different emotions for the conditions mask versus no mask, so we cannot keep the variance of depicted persons constant. Additionally, the study did employ only very few faces at all which always will increase the effectiveness of random effects caused by the idiosyncrasies of the utilized faces.

## The Present Study

The evident gap in knowledge of how face masks^1^ affect emotional reading in children made a specific study necessary, especially as the wearing of face masks has become a political issue (Wong, 2020) and the acceptance of masks is generally under risk (Egan et al., 2021). The topic of wearing masks is particularly emotionally and politically charged when it comes to children. Consequently, usage of face masks is highly debated for schools in particular (Spitzer, 2020). The major force behind aiming the present study was the second author (M. S.), a 9-year-old schoolboy from Florida, who contacted the first author (C.C.C.), who is a perceptual scientist with a focus on face research. After having read an article about C.C.C.’s specific research on emotional reading of faces in adults (i.e., Carbon, 2020) with masks typically used in the first wave of the COVID-19 Pandemic, M. S. was curious to know about the possibility to extend the study to a sample with school kids. He planned to conduct a replication study specifically with children because he identified this a valuable study as school kids strongly depend their everyday communication, especially in classrooms, on nonverbal communication—and even if this emotional reading might be limited in most cases to affirmations by expressing a happy face. M. S. also aimed at submitting such a replication study to the school’s STEM (Science, Technology, Engineering, and Mathematics) fair in 2020 (which he won—and, fortunately, he subsequently also won the second prize in the district STEM fair in 2020 with this project).

We decided to wholeheartedly collaborate on all issues of the scientific process in order to be able to submit a report about the study to the STEM committee of the school in time and by strictly following the usual scientific protocols. This required a strict organization and communication structure which we realized via the Open Science Framework (OSF). C.C.C. was in charge of guiding through the entire process, including explaining and teaching methodological as well as statistical basics, in order to present a conclusive and reliable scientific report. This short note of the entire collaboration is relevant, as we will briefly return to this important side kick of this research project later to make clear how STEM projects can attract school students by active involvement of scientific advisors and supervisors. All means towards the final product of a scientific paper were finely concerted with M. S.’s mother in order to optimally support M. S.

The main aim of the present study was to analyze the performance of reading basic emotions in faces that were masked versus unmasked. Here, we were particularly interested in gaining knowledge about the specific confusion of expressed emotions with the perception of these emotions. As the first author has conducted a similar study at the start of the first wave of the COVID-19 Pandemic with adults (Carbon, 2020), we were also keen to compare both datasets to gain knowledge on the specific problems children have with reading emotions from masked faces.

## Method

### Participants

Fifty-seven participants volunteered for the study (*M*_age_ = 9.7 years [9–11 years]; *N*_female_ = 28, *N*_male_ = 29); all of them were from elementary schools in Sarasota County, Florida, USA. Based on the comparison of Model #1 and Model #0, which directly tested the effect of face masking (see details in the Results section), we calculated the needed *N* via R package *simr* (Green & MacLeod, 2016). For both models to be compared we set the intercept to 60.0 which corresponds to an average performance of correctly recognizing the presented facial in 60% of the cases. For Model #1 we furthermore assumed the effect of face masks as a slope of –2.5 which corresponds to a decrease of correct recognition of emotions of 2.5% when faces with masks were presented—this rather small assumed slope was employed to be able to detect even small effect sizes with the targeted sample size. The random intercept variance was set to 10.0 and the residual standard deviation was set to 20.0; the α error level was set to 0.05. The desired test power (1–β) of 0.90 was approached with a minimum *N* = 57 (95%-CI of test power with 1,000 simulations: 0.88–0.92).

### Material

**Figure 1.**
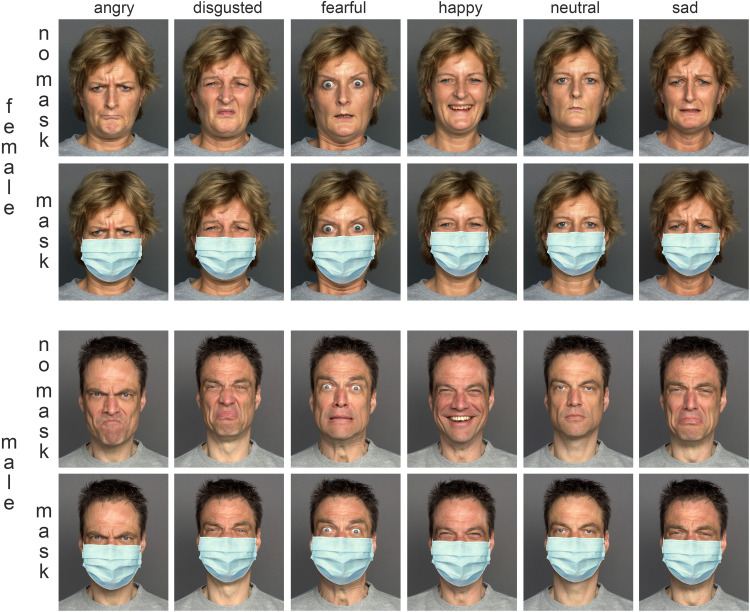
Two exemplary persons (a female in the upper part and a male in the lower part, both from the medium old age group) showing six different emotions without a mask (“no mask”) and wearing a mask (“mask”). Original material showing faces without face masks stems from MPI FACES database (persons #168 and #116, respectively, Ebner et al., 2010). Depiction used with kind permission of the Max Planck Institute—further distribution, publication or display beyond illustrating the research methodology of this study is prohibited by the Max Planck Institute.

All stimuli were based on frontal depictions of faces which were obtained from the MPI FACES database (Ebner et al., 2010) by a study-specific contract. Specifically, we employed frontal photos of four white European persons (previously called “Caucasian” in most research arenas), two female and two male, who belonged to two different face age groups (*young*: young persons #140 & #066 and *medium*: middle-aged persons #168 & #116—the hashtag numbers refer to the MPI FACES notation)—this range of young up to middle-aged adults was used to reflect the typical school setting with teachers of that age range in a school setting (but of course children also interact with people of different ages, for example, younger persons as peers, old people as grandparents). Six different pictures were used for each person that showed the emotional states angry, disgusted, fearful, happy, neutral, and sad. For the application of face masks to all of these 24 original pictures, we obtained a stock photo of a standard disposable mask in blue. The image of the mask was cut out via image processing software, which was then individually adapted to fit smoothly to the different face versions. This method offered the opportunity to use always the same face pictures but still showing a realistic way of mask-wearing. Figure 1 shows an exemplary female and male person from the middle-aged face age group used in the study.

In sum, we employed 2 [Face Sex] × 2 [Face Age Group] × 6 [Emotions] × 2 [Face Mask] = 48 facial versions in our study.

### Procedure

The experiment was set up as a Microsoft Forms project which was conveniently approachable via a QR code and which was distributed among participating school students. The entire study was conducted between 3 November 2020 (at 12:29 local time—Eastern Standard Time) and 19 November 2020 (at 10:06 EST) during the COVID-19 Pandemic, precisely, during the second rise of cases in the United States. Prior to the experimental session, written informed consent was obtained from the parent of each participant. Each participant was exposed to the complete set of stimuli. The stimuli were presented subsequently, with the order of trials being fully randomized across participants. The entire routine was repeated three times on consecutive days to gain more data points and to be able to check for training effects. Participants were asked to spontaneously assess the depicted person’s emotional state from a list of six emotions reflecting the same compilation of emotions shown by the different versions of the faces (*angry*, *disgusted*, *fearful*, *happy*, *neutral*, and *sad*). There was no time limit for giving a response. The general study design (psychophysical testing) was given ethical approval by the local ethics committee of the University of Bamberg. The entire procedure lasted approximately 3 × 8.5 ∼ 25 minutes. Afterwards, the participants were invited to be debriefed about the aims of the study, if wanted. Additionally, the study and its rationale was presented by the second author on the above mentioned STEM fair.

## Results

### Data Analysis Strategy

The data were processed using R 4.0.4 (R Core Team, 2021). In addition to the *lme4* package (Bates et al., 2015) to perform linear mixed effects analyses, R packages *lmerTest* (Kuznetsova et al., 2017) and *ggplot2* (Wickham, 2012) were mainly used during the analysis of the data. The entire anonymized dataset is available at the Open Science Framework (https://osf.io/4gt5r/?view_only=04623bd9c39d461b88fee3c37c250f21).

**Figure 2.**
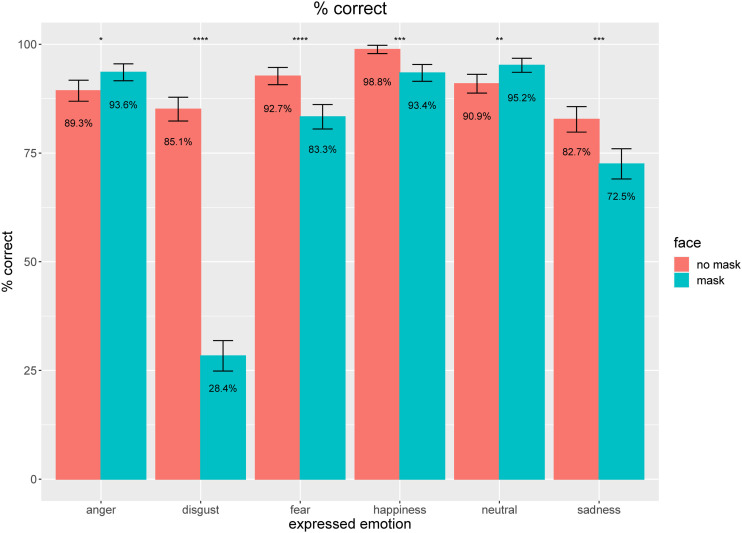
Mean percentage of correct assessment of the emotional states for faces with masks (blue) or without masks (red) on the face. Error bars indicate confidence intervals CI-95% based on adjusted values for taking within-subject variances into account (Morey, 2008). Asterisks indicate statistical differences between conditions of wearing and nonwearing on the basis of paired *t*-tests: *p < .05; **p < .01; ***p < .001; ****p < .0001. Highly similar results have been revealed by testing with Linear Mixed Models—see Table 2.

Overall performance for correctly identifying facial emotions in faces *without* masks was remarkable, *M* = 89.9% (chance rate: 1/6 = 16.7%), with average performance rates ranging across participants between 61.1% and 98.6%. As indicated by Figure 2, kids were particularly good at recognizing the emotional state happiness, fear, and neutral, followed by recognition performances, being still higher than 80%, for anger, disgust, and sadness. As soon as faces were covered by a typical blueish surgical mask, we detected an overall decrease of performance to 77.7% with average performance rates ranging across participants between 59.7% and 90.3%. We observed a pretty diverse pattern of performance changes from recognizing faces without masks to faces with masks: While we detected a dramatic drop in performance for disgust, the decreases for fear and sadness were still evident but less substantial (only about 10% of performance decrease). In addition, for happiness, the decline was only about 5%. Somehow unexpectedly, we also registered two emotions which showed *better* recognition performances: Anger and neutral showed an increase of performance by about 4%.

After having qualified the general pattern of data, we statistically tested the effect of wearing masks on the recognition of facial emotions by means of Linear Mixed Models (LMM). Our primary interest was in the impact of face masks on emotion recognition performance, so we first defined a null model (Model #0) with factors involved for which we had no specific hypothesis in mind: Model #0 used *session* (sessions 1–3) and *exprEmo* (*expressed emotion*: angry, disgusted, fearful, happy, neutral, and sad) as fixed effects and *caseID* (*participant*) as well as the *depictPers* (*depicted persons*: the four depicted persons used for the base faces) as random factors. Model #1 employed Model #0 as core and added *faceMask* (the presented face with a mask vs. without a mask) as a fixed factor. Model #2 added the interaction of *faceMask* and *exprEmo* as a further fixed factor following the idea that a face mask has a specific impact on the recognition of certain emotions which are mainly expressed by the facial information around the covered mouth-nose area. We always tested the more complex model with the preceding model, for instance, Model #1 against Model #0 via likelihood ratio tests. Each model’s residuals were visually inspected to exclude models deviating from homoscedasticity or normality. Table 1 shows this subsequent series of models, which identifies Model #2 as being the most adequate model concerning degree of fitting while being still parsimonious.

**Table 1.** Comparison of Models for the Dependent Variable Emotion Recognition Performance.

Model	*N_par_*	AIC	–2LL	*df*	χ^2^	*p*
#0: 1 + session + exprEmo + (1|depictPers) + (1|caseID)	11	81,072	–40,525			
#1: 1 + session + exprEmo + faceMask + (1|depictPers) + (1|caseID)	12	80,797	–40,387	1	275.6	<.0001
#2: 1 + session + exprEmo + exprEmo:faceMask + (1|depictPers) + (1|caseID)	17	79,952	–39,959	5	852.3	<.0001

*Note.* N_par_ = number of model’s parameters; AIC = Akaike information criterion, an estimator of prediction error; –2LL = likelihood ratio; *df*, *p* = degrees of freedom and *p*-value of the regarding χ^2^-test (comparing the present model with the preceding one, for example, the columns for Model #1 indicate the comparison between Model #1 and Model #0).

Table 2 shows the parameters of the finally selected Model #2 which explains 27.8% of the variance of the data. From session to session, participants earned higher recognition performance, indicated by significant effects of Session 2 and Session 3 tested against Session 1 (indicated by “Reference” in Table 2). Most importantly, we did not only find an overall effect of hampered emotional reading when masked faces were shown, but face masks had specific effects on certain facial emotions as demonstrated by significant interactions between expressed emotions and face mask wearing or not. We detected a particularly large effect of face masking on the reading ability of disgust, substantiated by an estimate of –56.73 for the interaction of *exprEmo* and *facemask* for the emotional state of disgust (see Table 2).

**Table 2.** Linear Mixed Model #2 Identified as Most Adequate to Describe the Data Pattern by Subsequent Testing of Model #1 Against Model #0 and Then Model #2 Against Model #1 via Likelihood Ratio Tests.

	Model #2		
*Predictors*	*Estimates*	*p*	*df*
(Intercept)	88.46***	**<0.001**	8,191.00
Session1	*Reference*		
Session2	2.78**	**0.001**	8,191.00
Session3	4.64***	**<0.001**	8,191.00
neutral	*Reference*		
anger	–1.61	0.342	8,191.00
disgust	–5.85***	**0.001**	8,191.00
fear	1.75	0.300	8,191.00
happiness	7.89***	**<0.001**	8,191.00
sadness	–8.19***	**<0.001**	8,191.00
exprEmoanger:faceMask	4.24*	**0.012**	8,191.00
exprEmodisgust:faceMask	–56.73***	**<0.001**	8,191.00
exprEmofear:faceMask	–9.36***	**<0.001**	8,191.00
exprEmohappiness:faceMask	–5.41**	**0.001**	8,191.00
exprEmoneutral:faceMask	4.24*	**0.012**	8,191.00
exprEmosadness:faceMask	–10.23***	**<0.001**	8,191.00
*ICC*	0.05		
*N*_depictPers |_ *N*_CaseID_	4 | 57		
Observations	8,208		
Marginal *R*^2^ / Conditional *R*^2^	0.243 / 0.278		
*AIC* | log-Likelihood	79,951.827 | –39,958.913		

*Note.* Bold numbers show significant results.

*p < 0.05. **p < 0.01. ***p < 0.001.

**Figure 3.**
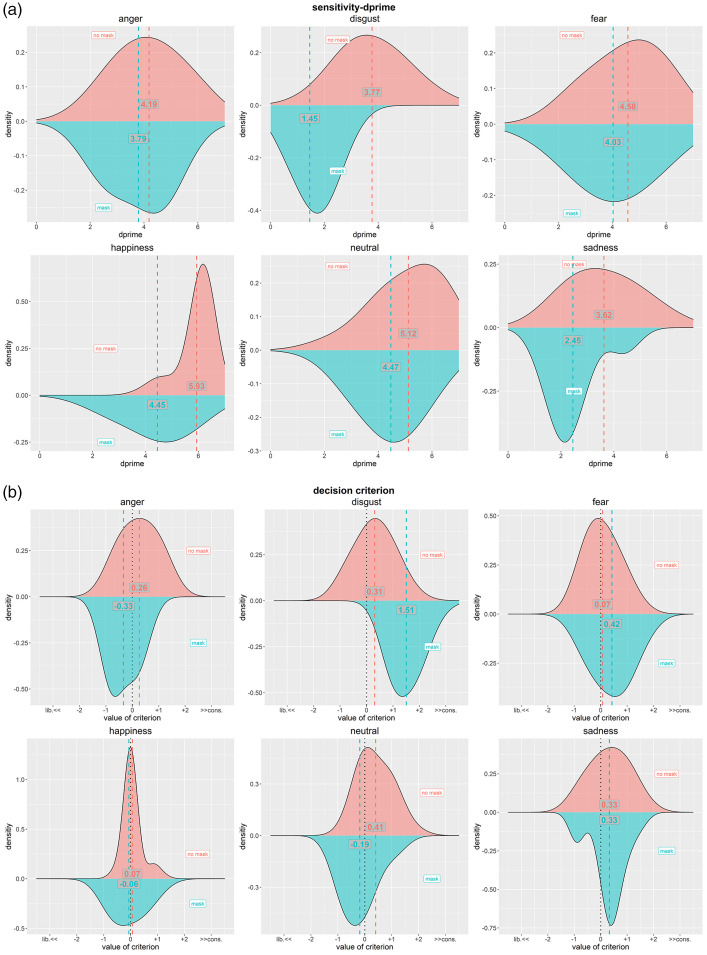
Analysis following a signal detection theory approach, conducted for each emotion separately: (a) sensitivity and (b) decision criterion. Upper data (red) in each sub figure show the condition with no masks, lower data (blue) show the condition with masks. Additionally, mean values are given as numbers along with vertical, colored lines. For (b) we also provide information on the quality of the criterion: Negative values indicate more liberal, positive values more conservative decision criteria; the neutral position (0) is further indicated by a dotted black vertical line.

Additionally, we followed a signal detection theory (SDT) approach to investigate whether the impact of face masks was mainly about the sensitivity of reading emotions or the response bias based on a different decision criterion. For conducting this additional analysis, we did not any more taking the sessions and different base faces into account. Figure 3 shows the respective distribution plus the means of the data for the sensitivity (operationalized by *dprime*, i.e., *z*_Hit_ – *z*_FA_) and the decision criterion (operationalized by *c*, i.e. –(*z*_Hit_ + *z*_FA_)/2), split by presentation conditions with and without masks. Adding a face mask had a clear main effect on reducing sensitivity (by 0.65) and changing the decision criterion by 0.59 towards a more liberal criterion, taking the neutral expression as reference level. More importantly, adding a face mask had a very different impact on specific emotions: Whereas fear was hardly affected by a mask, happiness, and disgust were particularly negatively impacted. The decision criteria for anger, fear, and neutral did not change very much (the respective change in the respective decision criterion *c* was always below 0.60) and for happiness and sadness, the change was even less pronounced. We obtained an evident change of decision criterion *c* for disgust only—see Figure 3.

We statistically tested the effect of wearing masks on the processing of facial emotions by means of independent LMM for the sensitivity measure *dprime* and the decision criterion *c*, respectively. Following the logic of the LMM above, we first defined a null model (Model #0) with factors involved for which we had no specific hypothesis in mind: Model #0 used *exprEmo* (*expressed emotion*: angry, disgusted, fearful, happy, neutral, and sad) as fixed factor and *caseID* (*participant*) as random factor. Model #1 employed Model #0 as core and added *faceMask* (the presented face with a mask vs. without a mask) as a fixed factor. Model #2 added the interaction of *faceMask* and *exprEmo* as a further fixed factor following the idea that a face mask has a specific impact on the recognition of certain emotions which are mainly expressed by the facial around of the covered mouth–nose area. For both dependent measures, we identified Model #2 as being the most adequate model concerning degree of fitting while being still parsimonious; see Table 3 for statistical details.

**Table 3.** Linear Mixed Model #2 Identified as Most Adequate to Describe the Data Pattern by Subsequent Testing of Models via Likelihood Ratio Tests.

	Model #2 (*dprime*)			Model #2 (*c*)		
*Predictors*	*Estimates*	*p*	*df*	*Estimates*	*p*	*df*
(Intercept)	5.12***	**<0.001**	670.00	0.41***	**<0.001**	670.00
neutral	*Reference*			*Reference*		
anger	–0.93***	**<0.001**	670.00	–0.14	0.215	670.00
disgust	–1.35***	**<0.001**	670.00	–0.10	0.404	670.00
fear	–0.54**	**0.005**	670.00	–0.33**	**0.004**	670.00
happiness	0.81***	**<0.001**	670.00	–0.34**	**0.003**	670.00
sadness	–1.50***	**<0.001**	670.00	–0.08	0.507	670.00
faceMask	–0.65***	**0.001**	670.00	–0.59***	**<0.001**	670.00
exprEmoanger:faceMask	0.26	0.349	670.00	–0.01	0.975	670.00
exprEmodisgust:faceMask	–1.66***	**<0.001**	670.00	1.79***	**<0.001**	670.00
exprEmofear:faceMask	0.10	0.710	670.00	0.94***	**<0.001**	670.00
exprEmohappiness:faceMask	–0.83**	**0.003**	670.00	0.46**	**0.005**	670.00
exprEmosadness:faceMask	–0.52	0.060	670.00	0.59***	**<0.001**	670.00
*ICC*	0.16					
*N*	57_CaseID_			57_CaseID_		
Observations	684			684		
Marginal *R*^2^/Conditional *R*^2^	0.491/0.573			0.340/NA		
*AIC* | log-Likelihood	2,092.397 | –1,032.199			1,305.184 | –638.592		

*Note.* Bold numbers show significant results.

*p < 0.05. **p < 0.01. ***p < 0.001.

**Figure 4.**
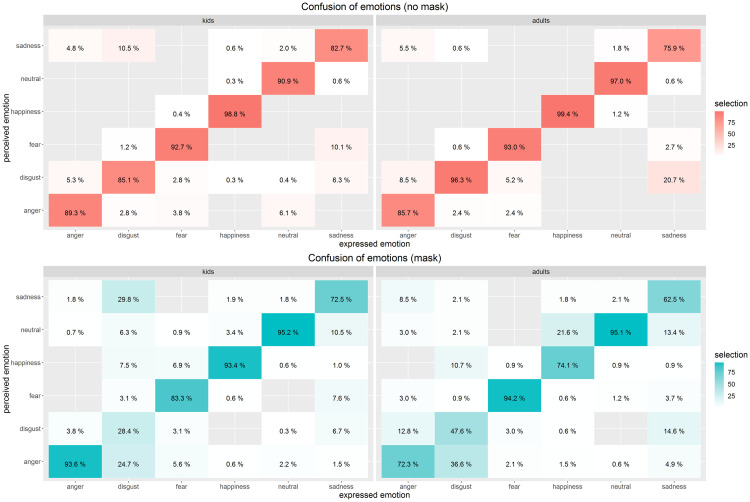
Confusion matrix of expressed vs. perceived emotions. Left side: relevant data of the present study; right side: comparison data from the study with adults from Carbon (2020)—note: the original Carbon study employed not only portraits of young and middle-aged persons, but also of elderly people; to be better able to compare the datasets of both studies we re-processed the Carbon (2020) dataset excluding the data for images of the elderly persons. Top matrices (in reddish hues): faces without masks, bottom matrices (in blueish hues): faces with masks. Percentages compile up to 100% for each expressed emotion. The more saturated the color, the higher the score of this cell. Empty cells indicate no perceived emotion of that kind, for example, expressed anger was never misperceived as happiness—for the conditions neither with nor without masking.

We were further interested in how specifically face masks affected the ability to read emotions regarding the confusion of emotions (specifically misperceiving an expressed emotion as a different one). As the confusion matrices in Figure 4 (left panel) show, participants were very good at perceiving the correct emotions as long as faces did not show face masks. Only for disgust and sadness, we observed characteristic misattributions of emotions in more than 10% cases specifically towards a specific alternative emotion: Disgust was misinterpreted as sadness in 10.5% of the cases and sadness was misinterpreted as fear in 10.1% of the cases. As soon as we added community masks to the depicted faces, participants showed stronger misinterpretations of emotions. Most pronouncedly, this happened for disgust which was nearly equally often perceived as sadness, anger, and disgust. Expressed sadness was much more interpreted as neutral when a mask covered the mouth area. Additionally, with a mask on, fear was often misinterpreted as happiness.

Although the overall pattern was relatively similar to the original study from May 2020 where we tested adult participants (Carbon, 2020), there are also important differences to be reported. As the original study with adults employed not only portraits of young and middle-aged persons, but also of elderly people, we re-processed the Carbon (2020) dataset excluding the data for images of the elderly persons in the following. As can be seen in Figure 4 (right panel), the control data of adult persons showed particular confusions for the emotions of sadness and disgust when masks were presented; however, for children, anger was even *more often detected* when a mask was present versus no mask was shown, whereas adults suffered a drop of performance in this respect. These dissociations warranted a deeper look into the data, so we decided to analyze the differences on basis of the SDT to be able to decide whether both groups differed primarily in terms of sensitivity or the decision criterion. In order to compare both datasets, we conducted two independent LMM, one for the sensitivity measure *dprime* and the other for the decision criterion *c*. The LMM which we employed (Model #C) contained *Study* (kids vs. adults), *faceMask* and *exprEmo* as fixed factors with full interaction among these factors, and *caseID* (*participant*) as the only random factor. Table 4 shows that kids showed overall lower scores for *dprime*, but higher scores for decision criterion *c* which indicate more conservative responses on average. However, we have to be cautious in interpreting main effects before analysing the interactive effects. We could indeed find dissociate patterns of the role of face masks, depending on the respective emotion: While kids showed higher sensitivity scores and more liberal response behavior for anger, they responded on disgusted faces in a more conservative response way.

**Table 4.** Testing the Effect of Age Group (Kids vs. Adults).

	Model #C-*dprime*			Model #C–*criterion*		
*Predictors*	*Estimates*	*p*	*df*	*Estimates*	*p*	*df*
(Intercept)	5.76***	**<0.001**	1150.00	0.11	0.240	1150.00
adults	*Reference*			*Reference*		
kids	–0.63**	**0.003**	1150.00	0.30*	**0.013**	1150.00
neutral	*Reference*			*Reference*		
anger	–1.86***	**<0.001**	1150.00	0.68***	**<0.001**	1150.00
disgust	–1.43***	**<0.001**	1150.00	–0.58***	**<0.001**	1150.00
fear	–0.68**	**0.003**	1150.00	0.15	0.232	1150.00
happiness	0.19	0.393	1150.00	–0.13	0.307	1150.00
sadness	–2.21***	**<0.001**	1150.00	0.55***	**<0.001**	1150.00
faceMask	–1.70***	**<0.001**	1150.00	–0.61***	**<0.001**	1150.00
faceMask:exprEmoanger	0.03	0.932	1150.00	0.20	0.265	1150.00
faceMask:exprEmodisgust	–0.88**	**0.006**	1150.00	1.98***	**<0.001**	1150.00
faceMask:exprEmofear	1.55***	**<0.001**	1150.00	0.40*	**0.031**	1150.00
faceMask:exprEmohappiness	–1.28***	**<0.001**	1150.00	1.31***	**<0.001**	1150.00
faceMask:exprEmosadness	0.87**	**0.007**	1150.00	0.90***	**<0.001**	1150.00
Studykids:exprEmoanger	0.92**	**0.002**	1150.00	–0.83***	**<0.001**	1150.00
Studykids:exprEmodisgust	0.08	0.781	1150.00	0.49**	**0.004**	1150.00
Studykids:exprEmofear	0.14	0.640	1150.00	–0.49**	**0.004**	1150.00
Studykids:exprEmohappiness	0.62*	**0.040**	1150.00	–0.21	0.228	1150.00
Studykids:exprEmosadness	0.71*	**0.018**	1150.00	–0.63***	**<0.001**	1150.00
Studykids:faceMask	1.04***	**<0.001**	1150.00	0.01	0.936	1150.00
Studykids:faceMask:exprEmoanger	0.23	0.585	1150.00	–0.21	0.384	1150.00
Studykids:faceMask:exprEmodisgust	–0.78	0.065	1150.00	–0.19	0.434	1150.00
Studykids:faceMask:exprEmofear	–1.45***	**0.001**	1150.00	0.55*	**0.023**	1150.00
Studykids:faceMask:exprEmohappiness	0.45	0.287	1150.00	–0.85***	**<0.001**	1150.00
Studykids:faceMask:exprEmosadness	–1.38**	**0.001**	1150.00	–0.31	0.202	1150.00
*ICC*	0.07					
*N*	57_CaseID_			57_CaseID_		
Observations	1,176			1,176		
Marginal *R*^2^/Conditional *R*^2^	0.554/0.586			0.379/NA		
AIC | log-Likelihood	3,518.555 | –1,733.278			2,137.115 | –1,042.557		

*Note.* The data of the adult participants stem from the dataset of Carbon (2020); for reasons of comparability, we only used the data for portraits with young and medium-aged persons. Linear Mixed Model #2 identified as most adequate to describe the data pattern by subsequent testing of models via likelihood ratio tests. Bold numbers show significant results.

*p < 0.05. **p < 0.01. ***p < 0.001.

## Discussion

We tested school kids aged 9 to 11 years on their recognition performance of facial emotions in the times of the COVID-19 Pandemic where face masks were common hygienic accessories to mitigate possible infections. The participants had to recognize emotional expressions displayed by faces which we showed with and without masks. The recognition performance was further qualified by comparing the data with a similar study that tested adult participants in May 2020 (Carbon, 2020).

First of all, the kids performed very well on a general basis. They reached nearly 90% of correct responses when confronted with faces without masks. This is quite remarkable as many theories claim a needed and ongoing maturation of face processing skills lasting about 12 to 14 years, particularly to develop the so-called *configural processing* mode (Mondloch et al., 2002; Schwarzer, 2006). Other researchers focusing on so-called *holistic processing* have found similar late maturation of expertise-based facial processing at an age between 11 and 15 years (Carbon et al., 2013), while other research indicated even longer periods needed to become a face expert, actually Germine et al. (2011) revealed in an extensive online study that learning abilities on faces peak after about an age of 30 years. When compared with the Carbon (2020) study employing adults with a mean age of 26.7 years ranging between 18 and 87 years, we see a highly comparable level of overall performance (for faces without masks: *M* = 89.5%). Even under the much information-restricted condition of recognizing emotions of masked faces, the kids’ overall performances were remarkably good (*M =* 77.7%), which was again comparable with adults’ overall performance—in fact, adults did even perform a bit less by about 5% (*M =* 72.7%). We also analyzed the data by means of a signal detection theory (SDT) approach. We revealed that face masks mainly impacted the sensitivity, but not so much the decision criterion of the children. Only the emotion of disgust was very much impacted by changing to a more conservative response behavior which means that children were less decisive in reporting the status of that emotional expression when a face mask was present.

When comparing our data with further studies on assessing the emotional status of faces, our sample of children also showed much better performances than the adult participants tested by Derntl et al. (2009) where performance rates of 73.2%, 73.7%, 63.2%, and 72.2% for sadness, anger, disgust, and fear, respectively, were detected. Derntl et al., however, employed less normative and clear stimuli and utilized a presentation limitation task, which should be taken into account when interpreting such performance figures in an absolute way. The overall performance finding is therefore more compatible with an early and fast cognitive development and maturation of face processing skills *sensu* McKone and colleagues who revealed that even young kids of only 4 to 5 years show qualitatively similar face perception skills as adults (McKone et al., 2009). When digging deeper into the underlying effects, we revealed a dissociative pattern of problems in the reading of facial emotions indicating *selective processes* which might be at work when recognizing emotions. This could be interpreted by nonunitary cognitive processing of emotions, which differs from standard models that assume general processing modes (Bruce & Young, 1986). While kids were nearly perfect in recognizing neutral and happy faces, they were very much handicapped when identifying disgust in faces presented with masks. In contrast, they even did better in detecting anger when faces were covered with masks. This could indicate that they more pronouncedly relied on the eyes region through which the emotional state of anger is mostly expressed. However, the clear drop of fear recognition, which is strongly expressed by eyes wide as saucers, does not support this view.

Previous studies identified the emotional states *happiness* and *sadness*, and to a lesser degree, also *anger*, as being mostly expressed by the lower facial part (Bassili, 1979; Fischer et al., 2012; Kret & de Gelder, 2012). Although exactly this area was covered by the presented face masks, we could only partly find a corresponding drop of performance. Actually, among these three focus candidates, only sadness was clearly affected by masking the mouth area. Compared with these emotions, we only detected mild negative effects in recognizing happiness. Most unexpectedly was the finding that the emotional state of anger and a neutral emotional state could even be *better* identified when face masks were present. These results were substantiated by respective significant interactions of these emotional states with *faceMask* (Table 2). In similar studies employing alternative means of covering the mouth area, it could be shown that anger was at least affected much less by occlusions through a rectangular cardboard (Bassili, 1979) or a niqāb (Fischer et al., 2012; Kret & de Gelder, 2012). There are also results which support the view that covering parts of the mouth can lead to better performances in certain tasks. By blocking out irrelevant or deceptive information in faces, people can sometimes focus on the relevant eyes region resulting in *better* performance (Kret & de Gelder, 2012)—to focus on the eyes region is also beneficial if people have to detect deception (Leach et al., 2016) which supports the view that some mental states are already fully detectable when observing only the eyes (Schmidtmann et al., 2020). The literature about the impact of occlusions on the ability to read facial emotions is all in all quite contradictory. For instance, angry faces are supposed to attract more attention to the eyes than the mouth (Eisenbarth & Alpers, 2011) which would be in line with the revealed data of the present study, but meanwhile Kotsia et al. (2008) showed that the occlusion of the mouth leads to lower detecting rates of anger. Our own finding of *superior* identification of anger in faces with masks shows that we have to investigate the employed methods in a much more differentiated way. We have to carefully analyze the specific stimuli, the selection of participants, the utilized paradigm and the interactive effects between participants and material. This also makes clear how important replicative studies are, especially if they start to decompose revealed effects into the underlying mechanisms and sources of variance.

Most notable in the present study is the strong negative effect of face masks on the recognition of disgust. Adult participants in an earlier, very similar study (Carbon, 2020) also showed a pronounced decline of performance for this specific emotion; however, adult participants mainly misperceived disgust as anger—reflecting a common finding (see, e.g., Cigna et al., 2015)—but they did not confuse disgust with sadness as the kids did. We do not yet know the base of this effect, but it points to the relevance of such findings for children’s everyday life: Face masks cover a large part of the face, and only so they seem to be effective in mitigating the viral load entering the respiratory passages through the mouth and the nose. This comes at a price, for example, an imposed change of the processing of faces. Changing processing does not evidently mean to impede the informative value of such processes. This can be tellingly seen with the emotional state of anger, which was better recognizable when faces were masked. Only the recognition of disgust was indeed dramatically affected, calling for effective and easy to implement countermeasures: In situations where disgust is aimed to be expressed, this should be accompanied by explicit and clearly pronounced verbal wording and, by nature and in daily routines already implemented, by a clear body language showing resistance and retreat (see Brotto et al., 2021). Mheidly et al. (2020) developed a sophisticated set of coping measures to enhance communication with face masks. In order to cope with hampered emotional reading, we can use the following actions from this set: (a) the increase of awareness of the typical communication challenges, (b) amplified utilizing of the upper face parts, (c) emphasizing body language, and (d) facing communication partners more directly and with more attention.

By all these thoughts, it is also clear that the application of masks to children should always be executed with much diligence and empathy. The usage of masks and the need to wear masks have to be comprehensibly explained (Esposito & Principi, 2020).

The present study tells us a further, important lesson: As we know, our capabilities as scientists to attract young people for STEM is rather limited, but explicit interest in our research expressed by children should be open-heartedly taken up. For fruitful, stimulating and productive interactions between STEM research and schools, it is important to take such interactions (like the present one where a 9-year-old schoolboy contacted a perceptual scientist) very seriously. We should support naturally interested school kids with the same level of commitment as typically invested in regular collaborations. Based on such a spirit, such collaborations will not be just promotionally effective events but will be serious scientific enterprises leading to new insights and potentially upcoming careers in the fields of STEM.

## Supplemental Material

sj-pdf-1-ipe-10.1177_20416695211038265 - Supplemental material for The Impact of Face Masks on the Emotional Reading Abilities of Children—A Lesson From a Joint School–University Project Click here for additional data file. Supplemental material, sj-pdf-1-ipe-10.1177_20416695211038265 for The Impact of Face Masks on the Emotional Reading Abilities of Children—A Lesson From a Joint School–University Project by Claus-Christian Carbon and Martin Serrano in i-Perception

## Note

1 We will use the term “mask” in a broad sense comprising typical accessories to cover the nose and the mouth used during the COVID-19 pandemics. These include nonprofessional protective items such as loop scarfs and home-made *community masks* as well as certified face masks such as FFP2/N95 masks. Note: Nonprotective /less-protective items like scarfs may be perceived differently than professional items of course (see Calbi et al., 2021).

## References

[bibr1-20416695211038265] Bassili J. N. (1979). Emotion recognition: The role of facial movement and the relative importance of upper and lower areas of the face. Journal of Personality and Social Psychology, 37(11), 2049–2058. 10.1037/0022-3514.37.11.2049 521902

[bibr2-20416695211038265] Bates D. Mächler M. Bolker B. Walker S. (2015). Fitting linear mixed effects models using lme4. Journal of Statistical Software, 67(1), 1–48. 10.18637/jss.v067.i01

[bibr3-20416695211038265] Biehl M. Matsumoto D. Ekman P. Hearn V. Heider K. Kudoh T., et al. (1997). Matsumoto and Ekman's Japanese and Caucasian Facial Expressions of Emotion (JACFEE): Reliability data and cross-national differences. Journal of Nonverbal Behavior, 21(1), 3–21. 10.1023/A:1024902500935

[bibr4-20416695211038265] Brotto D. Sorrentino F. Agostinelli A. Lovo E. Montino S. Trevisi P., et al. (2021). How great is the negative impact of masking and social distancing and how can we enhance communication skills in the elderly people? Aging Clinical and Experimental Research, 33(5), 1157–1161. 10.1007/s40520-021-01830-1 33725340 PMC7962629

[bibr5-20416695211038265] Bruce V. Young A. (1986). Understanding face recognition. British Journal of Psychology, 77(3), 305–327. 10.1111/j.2044-8295.1986.tb02199.x 3756376

[bibr6-20416695211038265] Calbi M. Langiulli N. Ferroni F. Montalti M. Kolesnikov A. Gallese V., et al. (2021). The consequences of COVID-19 on social interactions: An online study on face covering. Scientific Reports, 11, 2601. 10.1038/s41598-021-81780-w 33510195 PMC7844002

[bibr7-20416695211038265] Carbon C. C. (2020). Wearing face masks strongly confuses counterparts in reading emotions. Frontiers in Psychology, 11(2526), 1–9. 10.3389/fpsyg.2020.566886 33101135 PMC7545827

[bibr8-20416695211038265] Carbon C. C. Grüter M. Grüter T. (2013). Age-dependent face detection and face categorization performance. PlosOne, 8(10), e79164. 10.1371/journal.pone.0079164 PMC3792936 24116236

[bibr9-20416695211038265] Carragher D. J. Hancock P. J. B. (2020). Surgical face masks impair human face matching performance for familiar and unfamiliar faces. Cognitive Research-Principles and Implications, 5(1), 1–15. 10.1186/s41235-020-00258-x 33210257 PMC7673975

[bibr10-20416695211038265] Chu D. K. Akl E. A. Duda S. Solo K. Yaacoub S. Schunemann H. J., et al. (2020). Physical distancing, face masks, and eye protection to prevent person-to-person transmission of SARS-CoV-2 and COVID-19: Abrotto systematic review and meta-analysis. Lancet, 395(10242), 1973–1987. 10.1016/S0140-6736(20)31142-9 32497510 PMC7263814

[bibr11-20416695211038265] Cigna M.-H. Guay J.-P. Renaud P. (2015). La reconnaissance émotionnelle faciale: Validation préliminaire de stimuli virtuels dynamiques et comparaison avec les Pictures of Facial Affect (POFA) [Facial Affect Recognition: Preliminary Validation of Dynamic Virtual Stimuli and Comparison with Pictures of Facial Affect (POFA)]. Criminologie, 48(2), 237–263. 10.7202/1033845ar

[bibr12-20416695211038265] Cohen J. (1988). Statistical power analysis for the behavioral sciences (2nd ed.). Lawrence Erlbaum Associates. Hillsdale, NJ.

[bibr13-20416695211038265] Derntl B. Seidel E. M. Kainz E. Carbon C. C. (2009). Recognition of emotional expressions is affected by inversion and presentation time. Perception, 38(12), 1849–1862. 10.1068/P6448 20192133

[bibr14-20416695211038265] Ebner N. C. Riediger M. Lindenberger U. (2010). FACES-A database of facial expressions in young, middle-aged, and older women and men: Development and validation. Behavior Research Methods, 42(1), 351–362. 10.3758/brm.42.1.351 20160315

[bibr15-20416695211038265] Egan M. Acharya A. Sounderajah V. Xu Y. H. Mottershaw A. Phillips R., et al. (2021). Evaluating the effect of infographics on public recall, sentiment and willingness to use face masks during the COVID-19 pandemic: A randomised internet-based questionnaire study. BMC Public Health, 21(1), article number 367. 10.1186/s12889-021-10356-0 PMC7886844 33596857

[bibr16-20416695211038265] Eisenbarth H. Alpers G. W. (2011). Happy mouth and sad eyes: Scanning emotional facial expressions. Emotion, 11(4), 860–865. 10.1037/a0022758 21859204

[bibr17-20416695211038265] Esposito S. Principi N. (2020). To mask or not to mask children to overcome COVID-19. European Journal of Pediatrics, 179(8), 1267–1270. 10.1007/s00431-020-03674-9 32388722 PMC7210459

[bibr18-20416695211038265] Esposito S. Principi N. Leung C. C. Migliori G. B. (2020). Universal use of face masks for success against COVID-19: Evidence and implications for prevention policies. European Respiratory Journal, 55(6), 2001260. 10.1183/13993003.01260-2020 PMC7191114 32350103

[bibr19-20416695211038265] Fischer A. H. Gillebaart M. Rotteveel M. Becker D. Vliek M. (2012). Veiled emotions: The effect of covered faces on emotion perception and attitudes. Social Psychological and Personality Science, 3(3), 266–273. 10.1177/1948550611418534

[bibr20-20416695211038265] Freud E. Stajduhar A. Rosenbaum R. S. Avidan G. Ganel T. (2020). The COVID-19 pandemic masks the way people perceive faces. Scientific Reports, 10(1), 22344. 10.1038/s41598-020-78986-9 33349645 PMC7752904

[bibr21-20416695211038265] Germine L. T. Duchaine B. C. Nakayama K. (2011). Where cognitive development and aging meet: Face learning ability peaks after age 30. Cognition, 118(2), 201–210. 10.1016/j.cognition.2010.11.002 21130422

[bibr22-20416695211038265] Green P. MacLeod C. J. (2016). simr: An R package for power analysis of generalised linear mixed models by simulation. Methods in Ecology and Evolution, 7(4), 493–498. 10.1111/2041-210X.12504

[bibr23-20416695211038265] Kotsia I. Buciu I. Pitas I. (2008). An analysis of facial expression recognition under partial facial image occlusion. Image and Vision Computing, 26(7), 1052–1067. 10.1016/j.imavis.2007.11.004

[bibr24-20416695211038265] Kret M. E. de Gelder B. (2012). Islamic headdress influences how emotion is recognized from the eyes. Frontiers in Psychology, 3(110), 1–13. 10.3389/fpsyg.2012.00110 22557983 PMC3322610

[bibr25-20416695211038265] Kuznetsova A. Brockhoff P. B. Rune H. B. Christensen A. P. (2017). {lmerTest} Package: Tests in linear mixed effects models. Journal of Statistical Software, 82(13), 1–26. 10.18637/jss.v082.i13

[bibr26-20416695211038265] Leach A.-M. Ammar N. England D. N. Remigio L. M. Kleinberg B. Verschuere B. J. (2016). Less is more? Detecting lies in veiled witnesses. Law and Human Bmariniehavior, 40(4), 401–410. 10.1037/lhb0000189 27348716

[bibr27-20416695211038265] Marini M. Ansani A. Paglieri F. Caruana F. Viola M. (2021). The impact of facemasks on emotion recognition, trust attribution and re-identification. Scientific Reports, 11(1). 10.1038/s41598-021-84806-5 PMC7970937 33692417

[bibr28-20416695211038265] Matsumoto D. Ekman P. (1988). Japanese and Caucasian Facial Expressions of Emotion (IACFEE*)*. Intercultural and Emotion Research Laboratory, Department of Psychology, San Francisco State University.

[bibr29-20416695211038265] McKone E. Crookes K. Kanwisher N. (2009). The cognitive and neural development of face recognition in humans. In Gazzaniga M. (Ed.), The cognitive neurosciences (pp. 467–482). Cambridge, MA: Massachusetts Institute of Technology.

[bibr30-20416695211038265] Mheidly N. Fares M. Y. Zalzale H. Fares J. (2020). Effect of face masks on interpersonal communication during the COVID-19 Pandemic. Frontiers in Public Health, 8, 582191. 10.3389/fpubh.2020.582191 33363081 PMC7755855

[bibr31-20416695211038265] Mondloch C. J. Le Grand R. Maurer D. (2002). Configural face processing develops more slowly than featural face processing. Perception, 31(5), 553–566. 12044096 10.1068/p3339

[bibr32-20416695211038265] Morey R. D. (2008). Confidence intervals from normalized data: A correction to Cousineau (2005). Tutorials in Quantitative Methods for Psychology, 4(2), 61–64. 10.20982/tqmp.04.2.p061

[bibr33-20416695211038265] Nestor M. S. Fischer D. Arnold D. (2020). “Masking” our emotions: Botulinum toxin, facial expression, and well-being in the age of COVID-19. Journal of Cosmetic Dermatology, 19(9), 2154–2160. 10.1111/jocd.13569 32592268 PMC7361553

[bibr34-20416695211038265] Porschmann C. Lubeck T. Arend J. M. (2020). Impact of face masks on voice radiation. Journal of the Acoustical Society of America, 148(6), 3663–3670. 10.1121/10.0002853 PMC7857507 33379881

[bibr35-20416695211038265] R Core Team. (2021). *R: A language and environment for statistical computing*. http://www.R-project.org/

[bibr36-20416695211038265] Ruba A. L. Pollak S. D. (2020). Children’s emotion inferences from masked faces: Implications for social interactions during COVID-19. PLOS ONE, 15(12), e0243708. 10.1371/journal.pone.0243708 33362251 PMC7757816

[bibr37-20416695211038265] Schmidtmann G. Logan A. J. Carbon C. C. Loong J. T. Gold I. (2020). In the blink of an eye: Reading mental states from briefly presented eye regions. i-Perception, 11(5), 2041669520961116. 10.1177/2041669520961116 33088473 PMC7543157

[bibr38-20416695211038265] Schwarzer G. (2006). The development of face processing in infancy and early childhood: Current perspectives. American Journal of Psychology, 119(2), 329–334.

[bibr39-20416695211038265] Spitzer M. (2020). Masked education? The benefits and burdens of wearing face masks in schools during the current Corona pandemic. Trends in Neuroscience and Education, 20, 100138. 10.1016/j.tine.2020.100138 32917303 PMC7417296

[bibr40-20416695211038265] Stajduhar A. Ganel T. Avidan G. Rosenbaum R. Freud E. (2021, February 11). Face masks disrupt holistic processing and face perception in school-age children. *PsyArxiv*. 10.31234/osf.io/fygjq PMC8818366 35128574

[bibr41-20416695211038265] Verma S. Dhanak M. Frankenfield J. (2020). Visualizing the effectiveness of face masks in obstructing respiratory jets. Physics of Fluids, 32(6), 1–8. 10.1063/5.0016018 PMC7327717 32624649

[bibr42-20416695211038265] Wickham H. (2012). *ggplot2: Elegant graphics for data analysis*. Springer.

[bibr43-20416695211038265] Wong B. (2020, July 1). The psychology behind why some people refuse to wear face masks. *The Huffington Post.* https://www.huffpost.com/entry/psychology-why-people-refuse-wear-face-masks_l_5efb723cc5b6ca970915bc53

